# Effects of glucagon-like peptide-1 receptor agonists on psychiatric disorders: a systematic review

**DOI:** 10.1177/20451253251396304

**Published:** 2025-12-21

**Authors:** Serene Lee, Liyang Yin, Kayla M. Teopiz, Sabrina Wong, Gia Han Le, Naomi Xiao, Stephen Stahl, Kyle Valentino, Roger Ho, Melanie C. Zhang, Taeho Greg Rhee, Roger S. McIntyre

**Affiliations:** Brain and Cognition Discovery Foundation, Toronto, ON, Canada; Brain and Cognition Discovery Foundation, Toronto, ON, Canada; Brain and Cognition Discovery Foundation, Toronto, ON, Canada; Brain and Cognition Discovery Foundation, Toronto, ON, Canada; Department of Pharmacology and Toxicology, University of Toronto, ON, Canada; Brain and Cognition Discovery Foundation, Toronto, ON, Canada; Institute of Medical Science, University of Toronto, Toronto, ON, Canada; Department of Health Sciences, Queen’s University, Kingston, ON, CanadaBrain and Cognition Discovery Foundation, Toronto, ON, Canada; University of California, San Diego, San Diego, CA, USA; Brain and Cognition Discovery Foundation, Toronto, ON, Canada; Department of Pharmacology and Toxicology, University of Toronto, ON, Canada; Institute for Health Innovation and Technology (iHealthtech), National University of Singapore, Singapore, Singapore; Division of Life Science (LIFS), Hong Kong University of Science and Technology (HKUST), Hong Kong; Department of Psychological Medicine, Yong Loo Lin School of Medicine, National University of Singapore, Singapore, Singapore; Department of Psychiatry, University of Toronto, Toronto, ON, Canada; Department of Psychiatry, Yale School of Medicine, New Haven, CT, USA; Department of Public Health Sciences, University of Connecticut School of Medicine, Farmington, CT, USA; Department of Psychiatry, University of Toronto, ON, Canada Department of Pharmacology and Toxicology, University of Toronto, 77 Bloor Street West, Suite 617, Toronto, ON M5S 1M2, Canada

**Keywords:** Alzheimer’s disease, anxiety, depression, liraglutide, lixisenatide, Parkinson’s disease, substance use disorder

## Abstract

Extant literature pertaining to the administration of glucagon-like peptide-1 receptor agonists (GLP-1RAs) for Alzheimer’s disease, Parkinson’s disease, major depressive disorder, bipolar disorder, substance-, alcohol- and nicotine-use disorders, suggests promising efficacy beyond the current FDA-approved indications (e.g., type 2 diabetes mellitus, obesity). The implicated brain regions of the aforementioned mental disorders contain glucagon-like peptide 1 (GLP-1) receptors associated with improving cognitive and behavioral functioning. Therefore, we aimed to systematically review the treatment effects of GLP-1RAs in various neurocognitive and psychiatric disorders. Online databases including PubMed, OVID, MEDLINE, Embase, PsycINFO and Google Scholar, were searched from inception until October 1, 2024. Additional studies were identified from the reference lists of the included articles. 22 studies were identified, with a total of 186,847 participants included. Results reported that GLP-1RAs meaningfully improved cognitive and affective functioning (e.g., memory), which in some cases was sustained beyond exposure to the agent. Separately, multiple epidemiological studies reported that GLP-1RAs have protective effects, with a suggestion of decrease in the incidence of mental disorders. These results provides the impetus for large, long-term, randomized controlled trials for GLP-1 RAs for the treatment of various mental disorders. This review is not registered in PROSPERO or any other registry.

## Introduction

Glucagon-like peptide-1 receptor agonists (GLP-1RAs) are approved by the Food and Drug Administration (FDA) for the treatment of type 2 diabetes mellitus (T2DM), weight management in persons who are overweight (i.e., body mass index (BMI) ⩾ than 27 kg/m^2^) and related metabolic disorders.^1^ Additionally, GLP-1 RAs can mitigate obesity-related morbidity and reduce cardiovascular death (e.g., heart attack, stroke), in adults who are overweight or obese.^1^ The indications for GLP-1 RA use are similar but not identical across all agents.^1^ Preliminary evidence indicates that GLP-1 RAs may have treatment and/or protective effects for select mental disorders [Alzheimer’s Disease (AD), Parkinson’s Disease (PD), mood disorders as well as substance-, alcohol-, and nicotine-use disorders].^2–6^ In addition, persons with T2DM are disproportionately affected by neurological and psychiatric disorders compared to the general population, providing a compelling rationale for prioritizing GLP-1RAs as well as specifically evaluating GLP-1RAs as repurposed psychiatric drugs.^7,8^

GLP-1RAs are incretin mimetics that affect multiple physiological processes beyond glucose homeostasis and metabolism.^1^ GLP-1 is not only produced in L-cells of the gastrointestinal tract, but also in specific regions of the central nervous system (CNS) [e.g., nucleus tractus solitarius (NTS)].^6^ In addition, GLP-1 receptors are expressed throughout the CNS in brain structures implicated in the pathogenesis of AD and PD, such as the hippocampus and substantia nigra pars compacta (SNpc), respectively.^9,10^ Some GLP-1RAs (e.g., exenatide, lixisenatide, and liraglutide) are reported to cross the blood-brain barrier (BBB) and improve cognitive function, possibly by mediating measures of oxidative stress and neuroinflammation.^11–13^ In addition, GLP-1RAs have reported to inhibit neuroapoptosis and facilitate neuroprotection and neuroplasticity, providing molecular and cellular evidence of putative disease-modifying effects at a fundamental biological level.^11^

GLP-1RAs are also implicated in reward salience mechanisms, which may contribute to the weight-reducing effects of these agents as well as their protective and therapeutic effects in mental disorders.^14^ Herein, we synthesize published literature that has reported the effects of GLP-1RAs on mental disorders. The overarching aim is to review the therapeutic potential of GLP-1RAs as future treatment and prevention strategies for mental disorders.

## Methods

### Search strategy

A comprehensive search was conducted across online databases, including PubMed, OVID (including MEDLINE, Embase, and PsycINFO), and Google Scholar from inception until October 1, 2024. Subsequent manual searches of the reference lists of the obtained articles were conducted. The following Boolean search string was used: ((GLP-1) AND (human) AND ((“Parkinson's disease”) OR (“Alzheimer's disease”) OR (“bipolar disorder”) OR (depression) OR (anxiety) OR ("nicotine use disorder") OR ("alcohol use disorder"))). Studies were limited to the language of publication (e.g., English).

Two independent reviewers (SL, LY) screened the articles obtained using the Covidence software.^15^ After removing duplicates, articles were screened by title, abstract, and full text against the eligibility criteria (Table 1). Any discrepancies in screening between reviewers were resolved by discussion.

**Table 1.** Eligibility criteria.

Inclusion criteria 1. Clinical studies (e.g., randomized controlled trials, pilot studies, and observational cohort studies), 2. Participants must be administered with GLP-1RAs, 3. Participants must have one of the mental disorders (e.g., AD, PD, MDD, anxiety, BD, AUD, and NUD), 4. Comorbidities (e.g., T2DM and obesity) Exclusion criteria 1. Preclinical studies (e.g., animal studies), 2. Secondary data (e.g., systematic reviews, meta-analyses, narrative reviews, and post-hoc analyses), 3. Publication language not in English

AD, Alzheimer’s disease; AUD, alcohol use disorder; BD, bipolar disorder; GLP-1RAs, glucagon-like peptide-1 receptor agonists; MDD, major depressive disorder; NUD, nicotine-use disorder; PD, Parkinson’s disease; T2DM, type 2 diabetes mellitus.

### Data extraction

Extracted data was established a priori using a piloted data extraction table. Data extraction was conducted by two reviewers (SL, LY) and followed the 2020 Preferred Reporting Items for Systematic reviews and Meta-Analyses (PRISMA) guidelines in full. The extracted data included: (1) author(s) and publication year, (2) study design and participants, (3) intervention, (4) duration, and (5) outcome of interest(s) (Table 2; Supplemental Material).

**Table 2.** Descriptive characteristics of included clinical studies.

Author(s)	Participants	Intervention	Duration	Outcome(s) of interest
AD				
Gejl et al. (2016)	A total of 38 participants with AD were randomized to control (*n* = 20) or experimental (*n* = 18)	Liraglutide (0.6 mg) subcutaneously daily for 1 week, hereafter 1.2 mg daily for 1 week before increasing to 1.8 mg daily	26 weeks	Aβ deposition, glucose metabolic rate, and changes in cognitive capability, to which there was no significant difference in cognitive outcome within or between groups (*p* = 0.50). There were no significant differences in total cognitive score between groups (liraglutide 0.43, placebo 1.7; *p* = 0.50)
Gejl et al. (2017)	A total of 38 participants with AD were randomized to control (*n* = 20) or experimental (*n* = 18)	Liraglutide	6 months	Maximum glucose transport activity of the BBB to aggravate symptoms of AD. BBB glucose transport increased significantly in the experimental group during the first 6 months (*p* < 0.0001) but after 6 months, the groups no longer differed significantly (*p* = 0.24)
Mullins et al. (2019)	A total of 27 participants with AD were randomized to control or experimental. 18 participants completed the entire study	Exenatide (5 mcg) subcutaneously twice daily for 1 week, hereafter 10 mcg twice daily	18 months	Safety, tolerability, and adverse events, such as high incidence of nausea and decreased appetite. Neuropsychological measures were similar between the groups. However, the Digit-Span and maximum digit span forward were significant (*p* = 0.006, *p* = 0.017, respectively) at 6 months only. The visit factor revealed significant changes in the MMSE, Boston naming, and Digit-symbol total (*p* = 0.017, *p* = 0.038, *p* = 0.044, respectively)
Watson et al. (2019)	A total of 41 participants with subjective cognitive complaints were randomized to control (*n* = 16) or experimental (*n* = 25)	Liraglutide (0.6 mg in week 1, 1.2 mg in week 2, hereafter 1.8 mg) subcutaneously in the morning	12 weeks	Inverse correlation between IR and connectivity between the bilateral hippocampus. Increased connectivity in the experimental group, which extends findings of the negative effects of peripheral IR on the neural biomarkers of brain function
Edison et al. (2022)	A total of 204 participants with AD were randomized to control or experimental	Liraglutide subcutaneously daily	12 months	Temporal lobe, total gray matter, and frontoparietal volume were lower in the experimental group. Liraglutide-treated participants also showed a slower reduction in whole gray cortical matter, frontal, temporal, and parietal volume, and cognition
PD				
Aviles-Olmos et al. (2013)	A total of 45 participants with moderate PD were randomized to control (*n* = 24) or experimental (*n* = 21)	Exenatide subcutaneously daily	12 months	Exenatide was well-tolerated and showed clinically relevant improvements in PD across motor and cognitive measures. MDS-UPDRS scores improved by 2.7 points in the experimental group and declined by 2.2 points in the control (*p* = 0.037)
Athauda et al. (2017)	A total of 62 participants with moderate PD were randomized to control (*n* = 29) and experimental (*n* = 31)	Exenatide (2 mg) subcutaneously once weekly	48 weeks with a 12-week washout period	At 60 weeks, off-medication scores on part 3 of the MDS-UPDRS had improved by 1.0 points (95% CI: –2.6 to 0.7) in the experimental and worsened by 2.1 points (–0.6 to 4.8) in the control, an adjusted mean difference of –3.5 points (–6.7 to –0.3; *p* = 0.0318). Results were sustained beyond the period of exposure
Malatt et al. (2022)	A total of 63 participants with PD were randomized to control (*n* = 21) or experimental (*n* = 42)	Liraglutide (1.2 or 1.8 mg, as tolerated) self-administered injections daily	52 weeks	NMSS scores improved by 6.6 points in the experimental and worsened by 6.5 points in the control (*p* = 0.07). MDS-UPDRS part III and MDRS-2 score changes from baseline did not significantly differ
McGarry et al. (2024)	A total of 255 participants with early untreated PD were randomized to control (*n* = 85), experimental low-dose (*n* = 85), or experimental high-dose (*n* = 85)	NLY01 (2.5 or 5.0 mg)	36 weeks	At 36 weeks, 2.5 and 5.0 mg NLY01 did not differ from the control with respect to change in sum scores on MDS-UPDRS parts 2 and 3: difference versus placebo –0.39 (95% CI: –2.96 to 2.18; *p* = 0.77) for 2.5 mg and 0.36 (–2.28 to 3.00; *p* = 0.79) for 5.0 mg
Meissner et al. (2024)	A total of 156 participants with PD were randomized to control (*n* = 78) or experimental (*n* = 78)	Lixisenatide (10 μg for 14 days, hereafter 20 μg) subcutaneously daily before dinner	12 months with a 2-month washout period	At 12 months, scores on the MDS-UPDRS part III had changed by −0.04 points (indicating improvement) in the lixisenatide group and 3.04 points (indicating worsening disability) in the placebo group (difference, 3.08; 95% confidence interval, 0.86 to 5.30; *p* = 0.007)
Mood disorders (e.g., depression, anxiety, and bipolar disorder)				
Strawn et al. (2008)	A total of 16 participants that were healthy (*n* = 9) or had panic disorder (*n* = 7)	GLP-1RA (2 pmol/kg/min) intravenously	60 min	No individual experienced panic attacks during the study, and there were no significant changes in API scores following the infusion in either group. API scores changed from 1 ± 1 to 3 ± 5 and 0.2 ± 0.4 to 0 ± 0 in panic disorder participants and healthy participants, respectively
Mansur et al. (2017)	A total of 19 participants with MDD (*n* = 13) or BD (*n* = 6) and an impairment in executive function	Liraglutide (1.8 mg) daily adjunct to existing pharmacotherapy	4 weeks	Significant increase from baseline to week 4 in the TMTB standard score in the experimental (*p* = 0.009) and in composite *Z*-score comprising multiple cognitive tests (*p* < 0.001)
Gamble et al. (2018)	A total of 45,244 persons included in the observational cohort study using DPP-4i (*n* = 6206), sulfonylureas and DPP-4i (*n* = 22,128), GLP-1RA (*n* = 501), or GLP-1RA and sulfonylureas (*n* = 16,409)	DPP-4i, sulfonylureas and DPP-4i, GLP-1RA, or GLP-1RA and sulfonylureas	January 2007–January 2016	The incidence of depression or self-harm was 18.2 vs 13.6 events/1000 person-years in the GLP-1RA-cohort versus sulfonylureas, respectively. GLP-1RA was not associated with a significant change in incidence of depression or self-harm (GLP-1RA-cohort: unadjusted HR 1.36, 95% CI: 0.72–2.58; adjusted HR 1.25, 95% CI: 0.63–2.50)
Eren-Yazicioglu et al. (2021)	A total of 43 participants with T2DM and obesity were randomized to control (*n* = 20) or experimental (*n* = 23)	Exenatide (10 μg) twice daily	3 months	Stress scales (Childhood Trauma Questionnaire and Chronic Stress Scale, Cognitive Failures Questionnaire, and laboratory-based cognitive measures) were not statistically different between groups (*p* > 0.05)
Wium-Anderson et al. (2022)	Persons with T2DM (*n* = 116.699) and healthy persons (*n* = 116.008)	Insulin, metformin, sulfonylureas, and glinides combined, DPP4-i, GLP-1RAs, SGLT2i, or acarbose	2000–2012	Low doses of GLP-1RAs were associated with a lower risk of depression in persons with T2DM compared to nonusers (less than 3 μg of exenatide daily)
Battini et al. (2023)	Depressed persons experiencing therapy failure and depressed persons experiencing any other adverse event	Biguanides, sulfonylureas, thiazolidinediones, DPP4-i, GLP-1RAs, or SGLT2-i	FDA FAERS database (1967–2021) and WHO Vigibase (1968 to September 2021)	GLP-1RAs demonstrated the greatest potential protective effect, with statistically significant disproportionality scores (*p* = 0.000 and 0.033 from FAERS and Vigibase, respectively)
Tagliapietra et al. (2024)	Persons initiated on a GLP-1RA (*n* = 34,130) or DPP4-i (*n* = 105,478)	GLP-1RA or DPP4-i	June 1, 2013 to June 30, 2020	Incident depression occurred in 7.7 % (*n* = 2263) and 6.3 % (*n* = 6602) of GLP-1RA and DPP4-i users, respectively. The relative risk was 1.02, failing to demonstrate a significant increase in incident depression
AUD				
Klausen et al. (2022)	A total of 127 participants with AUD and alcohol dependence randomized to control (*n* = 65) or experimental (*n* = 62)	Exenatide (2 mg) subcutaneously once weekly, adjunct to standard cognitive-behavioral therapy	26 weeks	The number of heavy drinking days in both groups decreased with no significant differences (*p* = 0.46). However, in obese participants, exenatide reduced heavy drinking days by 23.6% (*p* = 0.034)
Probst et al. (2023)	A total of 151 participants with alcohol-consumption and smoking habits were randomized to control (*n* = 75) or experimental (*n* = 76)	Dulaglutide (Trulicy)	12 weeks	At week 12, participants in the experimental drank 29% less compared to the control (*p* = 0.04)
NUD				
Yammine et al. (2021)	A total of 84 prediabetic and/or overweight smokers were randomized to control (*n* = 42) or experimental (*n* = 42)	Extended-release exenatide (2 mg) subcutaneously once weekly adjunct to nicotine patch (21 mg) and smoking cessation counseling	6 weeks	Smoking abstinence increased in the experimental group compared to placebo (46.3% and 26.8%, respectively). WSWS scores did not reveal differential change over time between groups
Lengsfeld et al. (2023)	A total of 255 participants were randomized to control (*n* = 128) or experimental (*n* = 127)	Dulaglutide (1.5 mg) subcutaneously once weekly, adjunct to standard-of-care smoking cessation therapy	12 weeks	The biochemically confirmed 7-day point prevalence abstinence rate at 12 weeks was 27% in the experimental group compared to 18% in the control group
Luithi et al. (2024)	A total of 255 participants were randomized to control (*n* = 128) or experimental (*n* = 127)	Dulaglutide (1.5 mg) subcutaneously once weekly, adjunct to standard-of-care smoking cessation therapy	12 months	63% and 65% of experimental and control groups, respectively, were abstinent after 12 weeks. Abstinence rates declined to 43% and 41%, respectively, after 24 weeks (*p* = 0.11) and to 32% and 32%, respectively, after 52 weeks (*p* = 0.61)

AD, Alzheimer’s disease; AUD, alcohol use disorder; BD, bipolar disorder; GLP-1RAs, glucagon-like peptide-1 receptor agonists; IR, insulin resistance; MDD, major depressive disorder; MDRS-2, Mattis Dementia Rating Scale; MDS-UPDRS, Movement Disorder Society Unified Parkinson’s Disease Rating Scale; NUD, nicotine-use disorder; PD, Parkinson’s disease; T2DM, type 2 diabetes mellitus.

### Quality assessment

Study quality was assessed by two independent reviewers (SL, LY). Randomized controlled trials were assessed with the Cochrane Risk of Bias Tool for Randomized Studies (RoB2), and observational cohort studies were evaluated using the Quality Assessment Tool for Observational Cohort and Cross-Sectional Studies, adapted from the National Institute of Health (NIH) guidelines (Tables 3 and 4).^16,17^ Non-randomized studies with interventions were evaluated using the Robins-I Tool (Table 5).^18^

**Table 3.** Risk of bias assessment for randomized controlled trials.

Study	Item						Quality rating
	1	2	3	4	5	6	
Alzheimer’s disease							
Gejl et al. (2016)	L	L	L	L	L	L	Good
Gejl et al. (2017)	L	L	L	L	L	L	Good
Mullins et al. (2019)	M	L	L	L	L	L	Good
Edison et al. (2022)	M	M	L	L	L	L	Good
Parkinson’s disease							
Aviles-Olmos et al. (2013)	M	L	L	L	L	L	Good
Athauda et al. (2017)	L	L	L	L	L	L	Good
Malatt et al. (2022)	Full text unavailable						
McGarry et al. (2024)	L	L	L	L	L	L	Good
Meissner et al. (2024)	L	L	L	L	L	L	Good
Mood disorders (e.g., depression, anxiety, and bipolar disorder)							
Eren-Yazicioglu et al. (2021)	M	M	L	L	L	L	Good
Alcohol use disorder							
Klausen et al. (2022)	L	L	L	L	L	L	Good
Probst et al. (2023)	L	L	L	L	L	L	Good
Nicotine-use disorder							
Yammine et al. (2021)	L	L	L	L	L	L	Good
Lengsfeld et al. (2023)	L	L	L	L	L	L	Good
Luithi et al. (2024)	L	L	L	L	L	L	Good

H, high risk of bias; L, low risk of bias; M, medium risk of bias.

**Table 4.** Risk of bias assessment for observational cohort studies.

Study	Item														Quality rating
	1	2	3	4	5	6	7	8	9	10	11	12	13	14	
Mood disorders (e.g., depression, anxiety, and bipolar disorder)															
Gamble et al. (2018)	Y	Y	Y	Y	NR	Y	Y	NR	Y	Y	Y	NR	Y	Y	Good
Wium-Anderson et al. (2022)	Y	Y	Y	Y	NR	Y	Y	Y	Y	N	Y	NR	Y	Y	Good
Battini et al. (2023) - PDF	Y	Y	Y	Y	NR	NR	Y	NR	Y	N	Y	NR	Y	NR	Good
Tagliapietra et al. (2024)	Y	Y	NR	Y	NR	Y	Y	NR	Y	N	Y	NR	Y	Y	Good

CD, cannot determine; N, No; NA, not applicable; NR, not reported; Y, Yes.

**Table 5.** Risk of bias assessment for non-randomized studies with interventions.

Study	Item																								Quality rating
	1.1	1.4	1.5	1.6	1.7	1.8	2.1	2.4	3.1	3.2	3.3	4.3	4.4	4.5	5.1	5.2	5.3	6.1	6.2	6.3	6.4	7.1	7.2	7.3	
Mood disorders (e.g., depression, anxiety, and bipolar disorder)																									
Mansur et al. (2017)	N	Y	Y	N	Y	Y	N	Y	Y	Y	N	Y	Y	Y	Y	N	N	Y	Y	Y	Y	N	N	N	Good
	1.1	1.4	1.5	1.6	1.7	2.1	2.4	3.1	3.2	3.3	4.1	5.1	5.2	5.3	6.1	6.2	6.3	6.4	7.1	7.2	7.3				
Strawn et al. (2008)	N	Y	Y	N	N	N	Y	Y	Y	N	N	Y	N	N	PN	Y	Y	N	N	N	N		Good		

N, No; PN, Probably No; PY, Probably Yes; Y, Yes.

## Results

### Study results and selection

**Figure 1.**
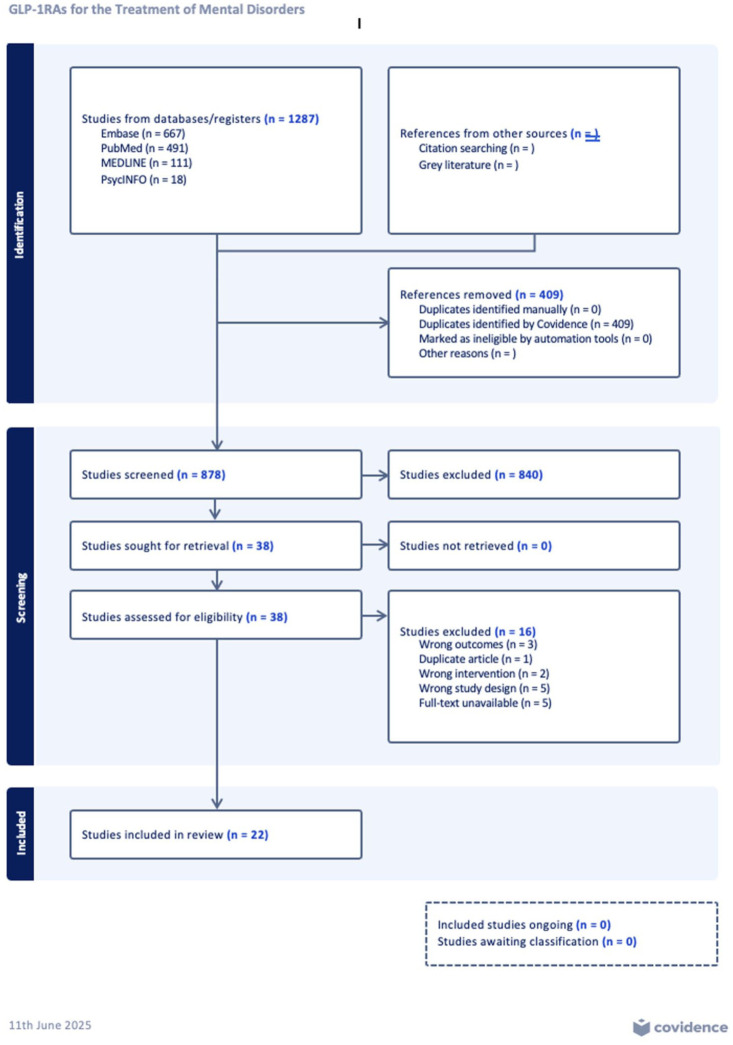
PRISMA flow diagram.

From the included databases and citation searching, 1287 studies were retrieved. After the removal of 409 duplicates, 878 studies remained for the title and abstract screening. A total of 38 full-text studies were assessed for eligibility based on the criteria, and 16 studies were excluded due to study design (*n* = 5), full-text unavailability (*n* = 5), wrong outcome(s) (*n* = 3), wrong intervention(s) (*n* = 2), and/or duplication (*n* = 1). Twenty-two studies were included in this systematic review (Figure 1).

### Clinical evidence of GLP-1RAs in AD

A total of five clinical studies were identified evaluating the effect of GLP-1RA therapy on AD. Gejl et al.^19^ and Gejl et al.^20^ reported the effects of liraglutide on participants (*n* = 38) with AD after 26 weeks. At 26 weeks, it was reported that there were no significant between- or within-group differences in either Aβ deposition as measured by positron emission tomography (PET) or cognitive performance, as measured by the Wechsler Memory Scale (WMS-IV) (*p* = 0.99 at baseline and *p* = 0.50 after treatment).^19^ During this study, the maximum glucose transport activity of the BBB with GLP-1RA therapy was monitored to establish the link between glucose metabolism and worsening of AD.^20^ Although early changes in glucose transport activity were noted, there were no significant differences between groups at endpoint.^19,20^ A negative correlation between AD progression and neuronal activity, as measured by glucose metabolism, was established, while a positive correlation between glucose metabolism and measures of cognition was noted (*p* = 0.01).^20^

A double-blind, randomized 18-month controlled pilot trial evaluated the efficacy, safety and tolerability of exenatide in participants living with AD (*n* = 18), (Mullins, Mustapic and Chia, 2019). A relatively high incidence of nausea and reduction in appetite were reported to be associated with GLP-1RA treatment (38% and 31%, respectively), which are well-described and common side effects associated with GLP-1RAs.^21^ In addition, measures of cognition were reported to be significantly improved in the exenatide-treated group at 6 months (e.g., Digit-Span *p* = 0.01, maximum digit span forward *p* = 0.02).^21^ Some improved cognitive measures were sustained over the course of the study, including the Mini-Mental State Exam (MMSE), Boston Naming, and Digit-symbol total (*p* = 0.02, *p* = 0.04, *p* = 0.04, respectively).^21^

In addition, Watson et al.^22^ reported on the effects of liraglutide on brain structures implicated in the pathogenesis of AD after 12 weeks, as well as examined the impact of insulin resistance (IR) preceding AD in participants (*n* = 41) with subjective cognitive complaints.^22^ Structural and functional magnetic resonance imaging prior to and after liraglutide administration revealed an inverse correlation between IR and connectivity in the bilateral hippocampus.^22^ The increased connectivity in the liraglutide group is consistent with the negative effects of IR on neural biomarkers of AD, raising the possibility that GLP-1RAs may play a protective role for individuals at risk for dementia.^22^ Similarly, Edison et al.^23^ also reported on brain structures implicated in PD following liraglutide administration for 12 months. They observed lower temporal lobe volumes, total gray matter, and frontoparietal matter in the liraglutide group.^23^ In addition, liraglutide-treated participants showed a slower reduction in whole gray cortical matter, the frontal, temporal, and parietal lobe volumes; changes in brain structural volume corresponded to improvement in measures of cognition.^23^

### Clinical evidence of GLP-1RAs in PD

A total of five clinical studies evaluating the effect of GLP-1RA therapy on individuals with PD were identified. Aviles-Olmos et al.^24^ and Athauda et al.^25^ both conducted randomized controlled trials administering exenatide subcutaneously and measured changes in the International Parkinson and Movement Disorder Society Unified Parkinson’s Disease Rating Scale (MDS-UPDRS). In the first study, exenatide was well-tolerated and showed clinically significant improvements in PD across motor and cognitive measures in participants (*n* = 45).^24^ After 12 months, MDS-UPDRS scores improved by 2.7 points in the experimental group and declined by 2.2 points in the control (*p* = 0.04).^24^ In the latter study, off-medication scores in MDS-UPDRS improved by 1.0 point (95% confidence interval (CI): –2.6 to 0.7) in the exenatide group and worsened by 2.1 points in the placebo group (95% CI: –0.6 to 4.8) at 60 weeks (*p* = 0.03).^25^ The effects of exenatide were sustained beyond the treatment period.^25^

A separate placebo-controlled study, Malatt et al.^26^ reported on the effects of liraglutide on the Movement Disorders Society Unified Non-Motor Symptoms Scale (NMSS), Mattis Dementia Rating Scale (MDRS-2), and MDS-UPDRS scores.^26^ After 52 weeks, NMSS scores improved by 6.6 points in the liraglutide group and worsened by 6.5 points in the placebo group (*p* = 0.07).^26^ However, changes in scores of the MDRS-2 and MDS-UPDRS did not significantly differ from baseline.^26^

Similarly, McGarry et al.^27^ and Meissner et al.^28^ reported the effects of an investigational GLP-1RA, NLY01, and lixisenatide, respectively, on MDS-UPDRS scores in participants with PD. McGarry et al.^27^ enrolled 255 participants with early untreated PD and observed nonsignificant changes in MDS-UPDRS scores between low-dose and high-dose NLY01 and placebo after 36 weeks (*p* = 0.77 for low-dose and *p* = 0.79 for high-dose). Meissner et al.^28^ enrolled 156 participants with PD, and observed significant changes in MDS-UPDRS scores between lixisenatide and placebo. After 12 months, there was a −0.04 point improvement in MDS-UPDRS scores in the lixisenatide group compared to a 3.04 point worsening in the placebo group (*p* = 0.01).^28^

### Clinical evidence of GLP-1RAs in mood disorders

A total of seven clinical studies evaluating the protective and treatment effects of GLP-1RAs on mood disorders [Major Depressive Disorder (MDD), anxiety or Bipolar Disorder (BD)] were retrieved from the search. Strawn et al.^29^ evaluated if GLP-1 infusions were associated with the induction of panic attacks and/or anxiety in healthy persons and persons with panic disorders (*n* = 9 and *n* = 7, respectively). It was observed that there were no significant changes in acute panic inventory (API) scores in either the GLP-1RA or placebo group (1 ± 1 to 3 ± 5 in panic disorder participants and 0.2 ± 0.4 to 0 ± 0 in healthy participants).^29^ A separate study by Mansur et al.^30^ reported on the effects of adjunctive liraglutide on executive function as measured by performance in the Trail-Making Test-B (TMTB). Participants (*n* = 19) with MDD or BD with preexisting impairment on executive function (i.e., 1 SD below the norm) were enrolled. From baseline to week 4, a significant improvement in TMTB scores in the liraglutide group was observed (*p* = 0.009).^30^ In addition, a significant increase in composite *Z*-score comprising multiple cognitive tests was reported (*p* = 0.001).^30^

The effects of GLP-1RAs in persons with depression were reported in several observational cohort studies conducted by Gamble et al.^31^, Wium-Anderson et al.^32^, Battini et al.^33^, and Tagliapietra et al.^34^ Gamble et al.^31^ observed a decrease in incident depression or self-harm in persons who had previously been prescribed dipeptidyl peptidase-4 inhibitors (DPP-4i), sulfonylureas, and DPP-4i, GLP-1RAs, or GLP-1RAs and sulfonylureas. The incidence of depression or self-harm was 18.2 versus 13.6 events/1000 person-years in the GLP-1RA-cohort versus sulfonylureas, respectively.^31^ While these trends suggest a possible association, the administration of GLP-1RAs was not associated with a significant change in incidence of depression or self-harm (unadjusted HR 1.36, 95% CI: 0.72–2.58; adjusted HR 1.25, 95% CI: 0.63–2.50).^31^

Similarly, Wium-Anderson et al.^32^ reported on the risk of incident depression in persons with T2DM previously prescribed insulin, metformin, sulfonylureas and glinides, DPP-4i, GLP-1RAs, sodium-glucose cotransporter-2 inhibitors (SGLT2i), and/or acarbose. Investigators observed GLP-1RAs, notably exenatide (not exceeding 3 μg daily), were associated with a lower risk of depression in persons with T2DM.^32^ Battini et al.^33^ reported on the potential protective effects of GLP-1RAs on depression, determining disproportionality scores from the FDA FAERS and WHO Vigibase. Among depressed persons experiencing therapy failure or any other adverse event, GLP-1RAs demonstrated significantly greater disproportionality scores compared to biguanides, sulfonylureas, thiazolidinediones, DPP4-i, and SGLT2-i (*p* < 0.001 and *p* = 0.03 from FAERS and Vigibase, respectively).^33^ Finally, Tagliapietra et al.^34^ compared incident depression in persons initiated on GLP-1RAs compared to DPP-4i. It was reported that depression occurred in 7.7% (2263/34,130) and 6.3% (6602/105,478) of users of GLP-1RAs and DPP-4i, respectively.^34^ These researchers reported a 1.02 relative risk of depression, thus failing to demonstrate a significant increase in incident depression.^34^ Results from retrospective cohort observational studies and pharmacovigilance reporting provide some suggestive evidence that GLP-1RAs may be associated with a lower risk for incident depression and/or suicidality, albeit results have been mixed and inconsistent, especially as it relates to pharmacovigilance studies. For example, the study conducted by Wium-Anderson et al. reported only a specific dose of exenatide (<3 μg daily) was associated with a lower risk of depression in persons with T2DM.

In addition, Eren-Yazicioglu et al.^35^ conducted a randomized controlled study of the cognitive and affective functioning effects of exenatide on participants (*n* = 43) with T2DM and obesity. Following 3 months of exenatide treatment, measures of stress and cognitive function as measured by multiple scales (e.g., Chronic Stress Scale and Cognitive Failures Questionnaire) were reported to not be statistically different between the exenatide and placebo groups.^35^ However, participants assigned to exenatide had higher Patient Health Questionnaire-9 scores (*p* = 0.03) and Perceived Stress Scale scores (*p* = 0.02), contributing to increased depression.^35^

### Clinical evidence of GLP-1RAs in substance use disorders

A total of five clinical studies evaluating the effects of GLP-1RAs on substance use disorders, including alcohol use disorder (AUD) (*n* = 2) and nicotine-use disorder (NUD) (*n* = 3), were identified from the search. Klausen et al.^36^ enrolled 127 participants with AUD and reported the effects of adjunctive exenatide and cognitive behavioral therapy on the number of heavy drinking days. At 26 weeks, the number of heavy drinking days in the exenatide and placebo group did not significantly differ.^36^ However, in participants who were obese (i.e., body mass index (BMI) ⩾ 30 kg/m^2^), the exenatide-treated participants evinced a significant reduction in heavy drinking days by approximately 24% (*p* = 0.03).^36^ A separate study conducted by Probst et al.^37^ enrolled 151 participants with alcohol-consumption and smoking habits (not AUD) to report the effects of dulaglutide on alcohol use. After study completion (i.e., 12 weeks), participants in the dulaglutide group evinced a significant reduction when compared to placebo in alcohol consumption (*p* = 0.04).^37^

The effects of GLP-1RAs in attenuating nicotine consumption, withdrawal and tolerance have been preliminarily evaluated. Yammine et al.^38^ conducted a pilot randomized controlled trial with 84 participants and reported on the effects of extended-release exenatide in combination with nicotine patches and smoking cessation counselling with nicotine abstinence as the primary outcome measure. At 6 weeks, exenatide in combination with nicotine replacement therapy (NRT) showed significantly greater smoking abstinence rates compared to persons receiving placebo and combination NRT.^38^ Exenatide also reduced end-of-treatment craving in the overall sample and withdrawal among abstainers.^38^ In addition, post-nicotine cessation weight gain was 5.6 lbs lower in the exenatide group compared to placebo (PP = 97.4%).^38^

Lengsfield et al.^39^ conducted an initial randomized controlled study, while Luithi et al.^40^ conducted a 12-month follow-up. In both studies, dulaglutide was administered as an adjunct to standard-of-care smoking cessation therapy in 255 participants. At 12 weeks, the 7-day point prevalence abstinence rate was 27% in the dulaglutide group compared to 18% in the placebo group.^39^ After 12 weeks, 63% and 65% of the dulaglutide and placebo groups, respectively, remained abstinent.^39^ At 24 weeks, abstinence rates declined to 43% and 41%, respectively (*p* = 0.11).^39^ Luithi et al.^40^ reported that at 12 months, abstinence rates in the dulaglutide and placebo groups were both 32%. Taken together, the aforementioned findings suggest GLP-1RAs may be effective in attenuating nicotine consumption in the acute phase; however, long-term efficacy and safety in this regard are not adequately characterized.

### Results from risk of bias assessments

Randomized controlled studies (*n* = 14) were evaluated using the Cochrane Risk of Bias Tool for Randomized Studies (RoB2) (Table 3). Among these studies, all were rated as “Good”; however, a few select studies had certain methodological limitations, including unclear selection and blinding processes.

Observational cohort studies (*n* = 4) were assessed using the Quality Assessment Tool for Observational Cohort and Cross-Sectional Studies, adapted from the NIH (Table 4). These studies received “Good” quality ratings with certain criteria not reported upon, including sample size justification and whether the outcome assessors were blinded to the exposure status of participants.

Finally, using the Robins-I Tool, both non-randomized studies with interventions were given “Good” ratings (Table 5). Whereas most criteria was met, the outcome assessors were aware of the intervention received by study participants, introducing a possibility of influence on cognitive outcomes.

## Discussion

GLP-1RAs show promise as potential treatment agents beyond the current FDA-approved indications for T2DM and obesity. Current clinical evidence has evaluated GLP-1RAs as therapeutic agents for several mental disorders, including AD, PD, MDD, anxiety, BD, AUD, and NUD. Results from the clinical studies indicate GLP-1RAs hold some promise as disease-modifying and/or treatments for several mental disorders, including AD and PD. This can be explained by the ability of exogenous GLP-1 to bind to GLP-1 receptors on brain regions implicated in disease pathogenesis, such as the hippocampus and SNpc.^9,10,41^ Cognitive function, as well as disease markers (e.g., Aβ plaque, α-synuclein), meaningfully improved in many of the aforementioned studies, suggesting GLP-1RAs as potential treatment agents.^9,10,19–28,41^

Results from clinical studies also indicate that GLP-1RAs have potential protective as well as treatment effects on mood disorders (e.g., MDD, anxiety, and BD).^29,35^ Moreover, several observational cohort studies reported that GLP-1RAs were not causally related to incident depression or self-harm, and that prior treatment of T2DM with GLP-1RAs consistently showed lower rates of incident depression compared to other treatment agents (e.g., DPP-4i and sulfonylureas).^31–34^ However, further investigation is needed to determine the reproducibility and clinical relevance as these findings are based on a limited number of studies with small sample sizes.

Preliminary clinical evidence evaluating the effects of GLP-1RAs for AUD and NUD indicate the potential efficacy in attenuating substance use, withdrawal, and maintaining abstinence.^38^ Overall, GLP-1RAs significantly increased abstinence rates and reduced withdrawal and craving symptoms, particularly in participants with obesity, though these outcomes were not consistently observed across all participants. Moreover, GLP-1RAs were associated with reducing post-nicotine weight gain and hyperphagia, which are common phenomena that promote return-to-smoking behavior.^38^ However, these findings should be interpreted cautiously due to the limited number of trials and small sample sizes.

The aforementioned findings are hypothesized to be subserved by modulation of reward salience mechanisms, notably the dopaminergic activity of the NTS, and the mesolimbic dopamine system.^42^ The expression of GLP-1 receptors on these brain regions provides a mechanistic explanation of how GLP-1RAs may regulate dopaminergic activity and reward sensations, which are upregulated in persons using alcohol and nicotine, predisposing them to addiction.^42^ There is also emerging cohort studies that have reported on the lower utilization of cannabis, tobacco, and opioid use in persons with GLP-1 RA prescriptions.^43,44^

GLP-1RAs have been insufficiently evaluated as therapeutic agents for mental disorders. Results herein indicate the potential protective and disease-modifying effects of GLP-1RAs.^40^ In addition, the potential benefits of GLP-1RAs reported in some circumstances extended beyond conventional metabolic targets, as observed in their potential for pain disorders.^45^

### Limitations

There are, however, several limitations that affect our analysis. The overarching limitation is the preliminary research investigating the effects of GLP-1RAs in bipolar disorder, anxiety disorder, and AUD. Many of the clinical studies administered GLP-1RAs adjunct to standard-of-care therapy, which makes it difficult to clearly determine the efficacy of GLP-1RA monotherapy. In addition, there are a few long-term studies that evaluate the effects of GLP-1RA therapy beyond the period of exposure, also limiting the observation of treatment-emergent adverse events.^39,40^ Finally, results from pharmacovigilance studies reported mixed results regarding the association of GLP-1 RAs and suicidality.^46,47^

## Conclusion

GLP-1RAs show promise as novel treatments in the disease courses of AD, PD, MDD, BD, AUD, and NUD. The pharmacologies of GLP-1 RAs and putative targeting in select brain regions implicated in the pathogenesis of the aforementioned mental disorders provides the compelling rationale for the reuporposing of GLP-1 RAs in the treatment and prevention of mental disorders.

## Supplemental Material

sj-docx-1-tpp-10.1177_20451253251396304 – Supplemental material for Effects of glucagon-like peptide-1 receptor agonists on psychiatric disorders: a systematic review Supplemental material, sj-docx-1-tpp-10.1177_20451253251396304 for Effects of glucagon-like peptide-1 receptor agonists on psychiatric disorders: a systematic review by Serene Lee, Liyang Yin, Kayla M. Teopiz, Sabrina Wong, Gia Han Le, Naomi Xiao, Stephen Stahl, Kyle Valentino, Roger Ho, Melanie C. Zhang, Taeho Greg Rhee and Roger S. McIntyre in Therapeutic Advances in Psychopharmacology

## Declarations

**Ethics approval and consent to participate:** Not applicable. This study is solely based on previously published literature and does not involve any human participants or identifiable data.

**Consent for publication:** Not applicable. This study does not include any individual data or images requiring consent for publication, as it is based entirely on published sources.

**Availability of data and materials:** The datasets generated and/or analysed during the current study are available in the PubMed repository, https://pubmed.ncbi.nlm.nih.gov/, and OVID repository, https://ovidsp.ovid.com/.
